# Are family physicians comprehensively using electronic medical records such that the data can be used for secondary purposes? A Canadian perspective

**DOI:** 10.1186/s12911-015-0195-x

**Published:** 2015-08-13

**Authors:** Karen Tu, Jessica Widdifield, Jacqueline Young, William Oud, Noah M. Ivers, Debra A. Butt, Chad A. Leaver, Liisa Jaakkimainen

**Affiliations:** Institute for Clinical Evaluative Sciences, G1 06, 2075 Bayview Avenue, Toronto, ON M4N 3M5 Canada; Department of Family and Community Medicine, University of Toronto, Toronto, Canada; University Health Network-Toronto Western Family Health Team, Toronto, Canada; Department of Family and Community Medicine-The Scarborough Hospital, Toronto, Canada; Women’s College Research Institute and Family Practice Health Centre, Women’s College Hospital, Toronto, Canada; Canada Health Infoway, Toronto, Canada; Department of Family and Community Medicine-Sunnybrook Health Sciences Centre, Toronto, Canada

**Keywords:** Electronic medical records, Adoption, Data completeness, Data quality

## Abstract

**Background:**

With the introduction and implementation of a variety of government programs and policies to encourage adoption of electronic medical records (EMRs), EMRs are being increasingly adopted in North America. We sought to evaluate the completeness of a variety of EMR fields to determine if family physicians were comprehensively using their EMRs and the suitability of use of the data for secondary purposes in Ontario, Canada.

**Methods:**

We examined EMR data from a convenience sample of family physicians distributed throughout Ontario within the **E**lectronic **M**edical **R**ecord **A**dministrative data **L**inked **D**atabase (EMRALD) as extracted in the summer of 2012. We identified all physicians with at least one year of EMR use. Measures were developed and rates of physician documentation of clinical encounters, electronic prescriptions, laboratory tests, blood pressure and weight, referrals, consultation letters, and all fields in the cumulative patient profile were calculated as a function of physician and patient time since starting on the EMR.

**Results:**

Of the 167 physicians with at least one year of EMR use, we identified 186,237 patients. Overall, the fields with the highest level of completeness were for visit documentations and prescriptions (>70 %). Improvements were observed with increasing trends of completeness overtime for almost all EMR fields according to increasing physician time on EMR. Assessment of the influence of patient time on EMR demonstrated an increasing likelihood of the population of EMR fields overtime, with the largest improvements occurring between the first and second years.

**Conclusions:**

All of the data fields examined appear to be reasonably complete within the first year of adoption with the biggest increase occurring the first to second year. Using all of the basic functions of the EMR appears to be occurring in the current environment of EMR adoption in Ontario. Thus the data appears to be suitable for secondary use.

## Background

Although North American countries have previously been lagging behind in the uptake of electronic medical records [EMRs] into their clinical practice [1], in recent years, with the introduction of government policies and mandates, physicians have been increasingly adopting EMRs into their clinical practice [2–5]. Canada Health Infoway strives towards the goal of one electronic health record for all Canadians, however in Canada, healthcare is organized at the provincial level and therefore each province has its own EMR adoption program and policies. Ontario was the first Canadian province to develop an EMR adoption program over a decade ago and although was initially slow in uptake [6], approximately 80 % of family physicians in Ontario are now on, or planning to go on an EMR in the near future [5]. Ontario’s EMR adoption program has targeted family physicians and provides physicians subsidization for adopting an approved EMR software into their clinical practice for capture of clinical activity that occurs within the family physician office and relevant information pertaining to the family physician management of patient health care. There are additional financial incentives if physicians can demonstrate that they are using their EMR to schedule patient appointments, enter encounter notes, enter problem lists, record prescriptions, generate automatic alerts/reminders to support care delivery and receive laboratory results electronically [7].

Government motivation to encourage EMR use includes expectations of both improvements in efficiency and quality of care. However evidence to support this notion is limited and whether or not EMR use results in improved patient care and outcomes is unclear [8–11]. Advanced features such as reminders, clinical decision support, and practice-based surveillance are considered to be amongst the most effective tools within the EMR to improve care [12–14] but cannot be used without having completed fields for the variables which trigger these tools. Understanding the extent to which physicians are using their EMRs is more important for improving care than simply assessing the presence or absence of an EMR. Previous studies that have looked at using electronic health record data for quality measurement [15] or comparative effectiveness research [16] have recognized the complexity of this type of evaluation and have provided conceptual frameworks on how to assess the data and issues to consider. Although useful in concept they do not provide real world analysis of the completeness of data. In addition a recent literature review assessing the reliability and validity of electronic health record quality measures found that most studies have been performed on data from large academic sites [17].

Few studies have looked at the time necessary to have reasonably complete EMR records. One study looked at data reliability but was confined to assessments for preventative services (pap smears, mammograms and influenza vaccinations). They found that data extracted from EMRs had limited reliability in the initial phase of EMR implementation but that during the second year after the introduction of the EMR, data reliability improved substantially [18].

It is unknown if the current government sponsored programs for EMR adoption including financial subsidies and peer leader support, are sufficient for supporting successful EMR implementation and it is unknown if physicians are comprehensively using their EMRs for all aspects of patient care. Evaluating the data quality of EMRs is not only important for policy makers and health administrators, but for researchers who seek to use EMRs for secondary purposes, such as research. Therefore, we evaluated the data population of a variety of EMR fields as a measure for completeness of EMR usage to assess if current policies are sufficient for EMR implementation and the suitability of the EMR data for secondary purposes. We also assessed the duration of time after EMR implementation to have complete EMR records and provide benchmarks for evaluation of the completeness of a variety of EMR fields.

## Methods

### Data source

We performed a retrospective review of data within the **E**lectronic **M**edical **R**ecord **A**dministrative data **L**inked **D**atabase (EMRALD) at the Institute for Clinical Evaluative Sciences [ICES]. EMRALD contains data from family physicians in Ontario that use Practice Solutions® EMR, the market leading EMR software vendor in Ontario, Canada [19]. All clinically relevant information from the EMRs are extracted. ICES is a ‘prescribed entity’ under provincial privacy legislation which provides the legal authority to collect individual level health information as it has the policies and procedures in place to protect patient privacy and confidentiality [20].

### Study participants

Data were extracted in the summer of 2012 from physicians who had been using their EMR for at least one year from both urban and rural locations distributed throughout Ontario. All physicians had a list of patients ‘rostered’ (enrolled) under them for which both the physician and the patient have a signed agreement with the provincial government identifying the physician that is responsible for the patient’s primary care service delivery [21]. The characteristics of physicians included in this study were compared to all family physicians in Ontario using the ICES Physician Database [IPDB].

### Patient population

Only patients that had a valid health insurance number, date of birth, and were rostered to the contributing physicians were included. Active patients were defined as rostered patients that had a physician visit within the one year prior to the date of data extraction or in the one year time interval examined. The characteristics of patients included in this study were compared to all patients rostered in Ontario and all Ontario residents. Neighborhood income quintiles and rural residence were determined through linking postal codes to Canadian census data [22]. General assessments of the burden of comorbidities were calculated using number of Ambulatory Care Groups (ACGs) [23] and presence of chronic conditions were determined using previously validated administrative data algorithms for identifying patients with specific disease conditions [24–27].

### Measures of EMR utilization

Measures were developed to evaluate the extent to which physicians were using the EMR to record their patient clinical encounters (visit documentation), blood pressures, weights, laboratory tests, prescriptions, referrals, specialist consultation letters and the population of all the fields of the cumulative patient profile (CPP). These EMR fields were grouped into *practice style independent fields* where the ideal situation would be 100 % completion (completed for every patient) and *practice style dependent fields* in which a lower level of utilization may not necessarily represent poor quality of EMR use but outlying physicians most likely represent poor users of the EMR for these functions (Table 1).

**Table 1:** Fields for EMR usage assessed and their measures

	EMR usage field	Measure^a^
Practice style independent fields	Visit documentation	Billings in the EMR for an office visit with a corresponding progress note entry on the same day
	Blood pressure and weight recording	Blood pressure or weight recorded in the structured variable
	Completeness of the Cumulative Patient Profile [CPP]	Populated allergies, immunizations, active treatment, risk factors, personal traits, family history and medical history [history of past health and problem list included together] fields
Practice style dependent fields	Laboratory test results provided through an electronic link.	At least one laboratory test
	Using the EMR to generate and record prescriptions	At least one prescription
	Using the EMR for generating referral letters from the family physician to the specialist	Entry in the EMR in the referral’s field
	EMRs including specialist consultant reports.	Entry in the EMR in the specialist’s consulatation letter field

^a^Measures calculated as the mean percent for number of active rostered patients [rostered patients with completed fields for CPP measures] having the measure in a given year/the number of active rostered patients [rostered patients for CPP measures] in a given year

Blood pressures and weights in the EMR are automatically captured in structured variable fields if entered using the nomenclature bp: and wt: respectively. The advantage for clinicians of having structured variable fields is that the measures can be searched and graphed to see changes over time. The advantage for researchers is that these measures can easily be identified and analysed. Physicians that have low recordings of these measures may not have been doing these measures, not recording these measures, or not recording these measures with the proper nomenclature such that the measurement populated the structured variable field.

Laboratory results can come into the EMR either through an automated electronic feed from the laboratory, manually entered in, or scanned into the EMR as a report. Only laboratory results entered into the EMR in a structured variable field, as what occurs in the instance of an automated electronic feed, are readily accessible both for analysis and for clinical care. Information stored in a structured variable field can be quickly searched and graphed to look at trends over time whereas information in scanned documents tends not to be searchable.

Some physicians who are not fully using their EMRs may opt to write their prescriptions on a prescription pad or a referral on a paper referral form. However doing so would result in incomplete EMR patient records and may not allow for use of advanced EMR functions. Thus we assessed the percent of active patients that had a prescription or referral letter. Physicians with really low numbers of ‘active’ patients with a prescription or a referral letter were thought to be using paper to perform these functions rather than their EMR.

Currently there is no province-wide program that facilitates automatic electronic transfer of consultation letters from specialists and hospital discharge summaries. Mechanisms to electronically transfer hospital discharge summaries are now starting to roll out but at the time of the study these mechanisms were not in place. We therefore looked at the number of consult letters compared to the number of rostered patients, as an approximate measure, to see if these external documents that were coming into physician offices were being entered into the EMR.

The CPP includes the categories: family history, medical history including history of past health and problem list (a list of current active problems), allergies, immunizations, active treatments, risk factors and personal traits. Within the EMR there are two separate fields for the history of past health and for a list of active problems. These two fields were grouped together since physicians do not use the two separate fields in a consistent manner.

### Measuring time on EMR

Completeness of EMR fields by *physician time on EMR* was assessed by looking at each year since initiation of the EMR. The initiation date of the EMR was defined as the earliest date with at least 10 progress notes with a corresponding bill for a visit that were recorded on the same day. Only physicians that had a full year of data in the relevant year were included in the denominators for assessment. For example only the patients of the physicians that had at least 4 years of data were included in the assessment of the completeness of the EMR fields for the physician time on EMR 4 year category. The analysis for *patient time on EMR* was done by assessing both the EMR fields and the CPP fields in the one year prior to the most recent date of data extraction for patients that had a record in the EMR for <1 year, 1-2 years, 2-3 years and >3 years. Patient time on the EMR was calculated from first date of a progress note and a physician bill on the same date to the date of the most recent data extraction. The analysis for the EMR fields for the *patient time on EMR* were confined to just those patients that were ‘active’ (had a visit in the last year since extraction).

### Statistical analysis

Physician characteristics by duration of EMR use were descriptively analyzed. We estimated the average completeness by duration of EMR use for each field with 95 % confidence intervals [CI] constructed around the average frequency of data completeness. Analyses were performed using SAS version 9.2 [SAS Institute, Cary, North Carolina].

### Setting benchmarks

We looked to identify benchmarks of completeness for each of the EMR fields, as benchmarks of this type have not previously been reported. The EMRALD team including several family physicians/EMR users discussed and agreed upon benchmarks for optimal completeness. The rationale behind these benchmarks included considerations for clinical use and secondary use of EMR data.

For the *practice style independent fields* we agreed that visit documentation (documentation for the purposes of clinical record keeping) and allergies were clinically the most important for patient safety and thus a benchmark of 95 % data completeness was set. For the other *practice style independent fields* a benchmark of 80 % completeness was agreed to be sufficient, feasible and clinically meaningful. For *practice style dependent fields* we recognized that physicians may simply prescribe fewer medications, order fewer lab tests, or refer their patients less than their colleagues. Thus it was decided that benchmarks for these parameters was best suited to be data driven, to identify outliers with really low population of these fields potentially indicating systemic EMR issues rather than physician practice style issues. The benchmark for these fields was set at the mean for the measure less one standard deviation.

Physicians were plotted on frequency distribution graphs and benchmarks were drawn to gain an understanding of where physicians lay in terms of meeting benchmarks.

This study received ethics approval from the Sunnybrook Health Science Centre Research Ethics Board. Individual level physician consent was not required for this study. As a prescribed entity under Ontario’s *Personal Health Information Protection Act*, ICES is authorized to collect and use personal health information from health organizations and clinics without consent for the purposes of health system evaluation and monitoring. Additionally, ICES is prohibited, under its agreements with data providers, from contacting individuals whose information has been entrusted to ICES. This contractual obligation restricts any opportunity to seek individuals’ consent for use of their information.

## Results

Although the patients included in this study were more from higher income quintiles and living in rural locations, the age and sex, presence of chronic conditions and measures of comorbidity were similar to rostered patients in Ontario (Table 2). There were differences in all the characteristics when comparing EMRALD participating physicians to the rest of the primary care physicians in Ontario (Table 3). Physician characteristics by duration of EMR use are reported in Table 4. Of the 167 physicians with at least one year of EMR use, we identified 186,237 patients. The number of physicians, and the corresponding number of patients, decreased with increasing physician time of EMR use.

**Table 2:** Comparison of EMRALD study cohort patients, rostered patients in Ontario and all residents of Ontario as of March 31, 2012

	Study cohort	Rostered Ontario patients	All Ontario residents
Number of people	185734^a^	10,230,063	14,005,291
Sex			
Male	46.2 %	47.5 %	49.1 %
Female	53.8 %	52.5 %	50.9 %
Age Groups			
0-17	18.9 %	18.2 %	20.6 %
18-29	14.2 %	15.1 %	16.2 %
30-44	19.8 %	20.3 %	20.9 %
45-64	30.0 %	30.2 %	28.0 %
65-84	14.6 %	14.1 %	12.4 %
85+	2.4 %	2.1 %	1.9 %
Unknown	0.2 %	0.0 %	0.0 %
Mean age	41.3 years	41.2 years	39.1 years
Neighborhood Income Quintile			
1 - Lowest Income	15.7 %	17.7 %	18.8 %
2	17.8 %	19.2 %	19.1 %
3	19.2 %	20.2 %	19.6 %
4	21.6 %	21.7 %	20.7 %
5 - Highest Income	24.8 %	20.7 %	19.8 %
Unknown	0.8 %	0.4 %	2.0 %
Rurality			
Rural	27.3 %	11.6 %	11.0 %
Urban	72.3 %	88.3 %	87.3 %
Unknown	0.5 %	0.1 %	1.7 %
Number of Adjusted Clinical Groups [ACGs]^b^			
0	6.3 %	6.2 %	9.4 %
1	47.4 %	41.8 %	41.1 %
2	37.2 %	40.6 %	36.2 %
3	7.9 %	10.0 %	8.8 %
Unknown	1.1 %	1.4 %	4.6 %
Chronic Conditions^b^			
Any chronic condition	52.8 %	55.9 %	50.8 %
Previous Acute Myocardial Infarction	1.9 %	1.7 %	1.5 %
Asthma	12.9 %	14.0 %	12.9 %
Congestive Heart Failure	2.3 %	2.2 %	2.0 %
Chronic Obstructive Pulmonary Disease	7.9 %	8.1 %	7.2 %
Diabetes	10.2 %	12.4 %	11.0 %
Hypertension	26.2 %	28.5 %	25.2 %
Mental health issue	20.8 %	22.8 %	20.9 %

Note: Percentages may not add to 100 due to rounding

^a^Some study cohort patients could not be linked to the administrative databases due to changing health card numbers

^b^Number of ACGs and chronic conditions calculated using ICES validated administrative data algorithms only for people over 18 years of age; chronic conditions are not mutually exclusive

**Table 3:** Comparison of study cohort physicians and all primary care physicians in Ontario as of March 31, 2012

Characteristic	EMRALD cohort physicians		All primary care physicians in Ontario^a^	
	N	%	N	%
Sample Size	167	100.0	8054	100.0
Sex				
Female	94	56.0	3333	41.4
Male	74	44.1	4721	58.7
Age group				
Under 35 years	25	14.9	500	6.2
35-44 years	57	33.9	1643	20.4
45-54 years	36	21.4	2425	30.1
55-79 years	46	27.4	3471	43.1
Unknown	4	2.4	15	0.2
Medical training location				
Canada	150	89.3	5967	74.1
International [including US]	17	10.1	2074	25.8
Unknown	1	0.6	13	0.2
Rurality				
Rural	32	19.1	631	7.6
Suburban	39	23.2	1355	16.3
Urban	97	57.7	6325	76.1
Visits in the Emergency Department				
More than 25 % of *practice/bills*	20	11.9	347	4.3
Less than 25 % of *practice/bills*	148	88.1	7707	95.7
Practising in a patient enrolment model group				
Full time affiliation	157	93.5	6774	84.1
Not affiliated	11	6.6	1280	15.9
	Mean	Range	Mean	Range
Physician Age on March 31, 2012	46.6	28-69	52.2	27-79
Years in practice	15.2	1-36	18.5	0-45
Years since graduation	19.9	3-43	26.3	2-65

^a^Primary care physicians were defined as having a main speciality of General Practitioner/Family Physician or Community Medicine/Public Health who’s practice is focused on primary care

**Table 4:** Physician characteristics by duration of EMR use

Characteristic	Duration of EMR use				
	At least 1 year	At least 2 years	At least 3 years	At least 4 years	At least 5 years
Number of physicians	167	145	132	92	85
Percent female physicians	55.7 %	54.5 %	52.3 %	48.9 %	48.2 %
Mean physician age in years [SD]	45.3 [10.4]	46.0 [10.3]	46.7 [10.1]	47.3 [10.1]	47.7 [9.9]
Mean number of years in practice in Ontario [SD]	14.3 [-9.3]	15.1 [-9.0]	15.6 [-8.7]	15.9 [-8.4]	16.5 [-8.2]
Percent in rural practice location	18.0 %	20.0 %	21.2 %	17.4 %	17.7 %
Mean duration of EMR use in years [SD]	5.0 [-3.1]	5.6 [-3.0]	5.9 [-3.0]	7.0 [-2.9]	7.2 [-2.9]
Total number of patients	186,237	165,040	151,072	112,521	104,985

**Fig. 1:**
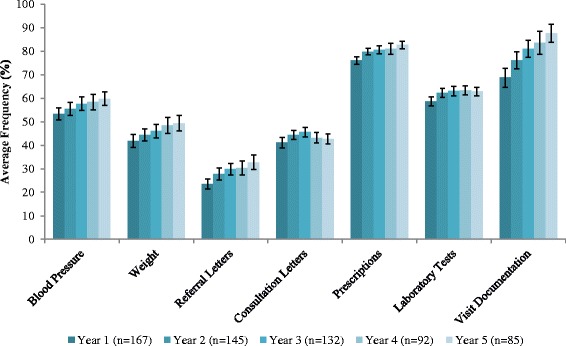
Average completeness by physician’s duration of EMR use for the fields: visit documentation, blood pressures, weights, laboratory tests, prescriptions, referrals and consultation letters

For the physician time on EMR analysis, the completeness by physician’s duration of EMR use for each field (visit documentation, blood pressures, weights, laboratory tests, prescriptions, referrals and consultation letters) are illustrated in Fig. 1. Overall, the fields with the highest level of completeness for the average proportion of the number of patients were for visit documentation and for prescriptions (≥70 %). By duration of physician EMR use, the average completion of visit documentation increased from 69 % [95 % CI 65-73] in Year 1 to 88 % [95 % CI 84-92] by Year 5. Over time, increasing trends of completeness for all fields were observed, except for laboratory tests and consultation letters.

**Fig. 2:**
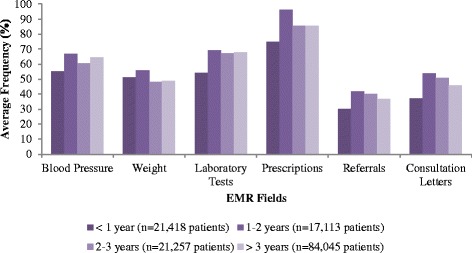
Population of various fields as a function of patient duration on the EMR

**Fig. 3:**
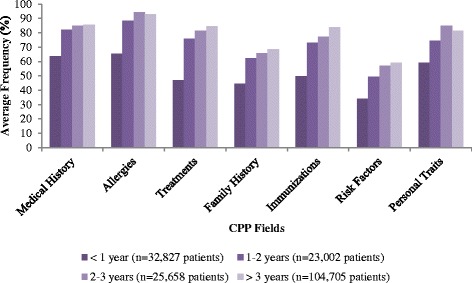
Population of the cumulative patient profile [CPP] fields as a function of patient duration on the EMR

For patient time on the EMR there was an increasing population of blood pressure, laboratory tests and prescriptions over time. No increases were observed for documentation of patient weights, referrals and consultations for patients that had been on the EMR for over 3 years (Fig. 2). For population of the CPP fields, there was an increasing trend for completeness of all fields except for allergies and personal traits for patients with >3 years of patient data on the EMR (Fig. 3). The largest improvements occurred between one and two years.

**Fig. 4:**
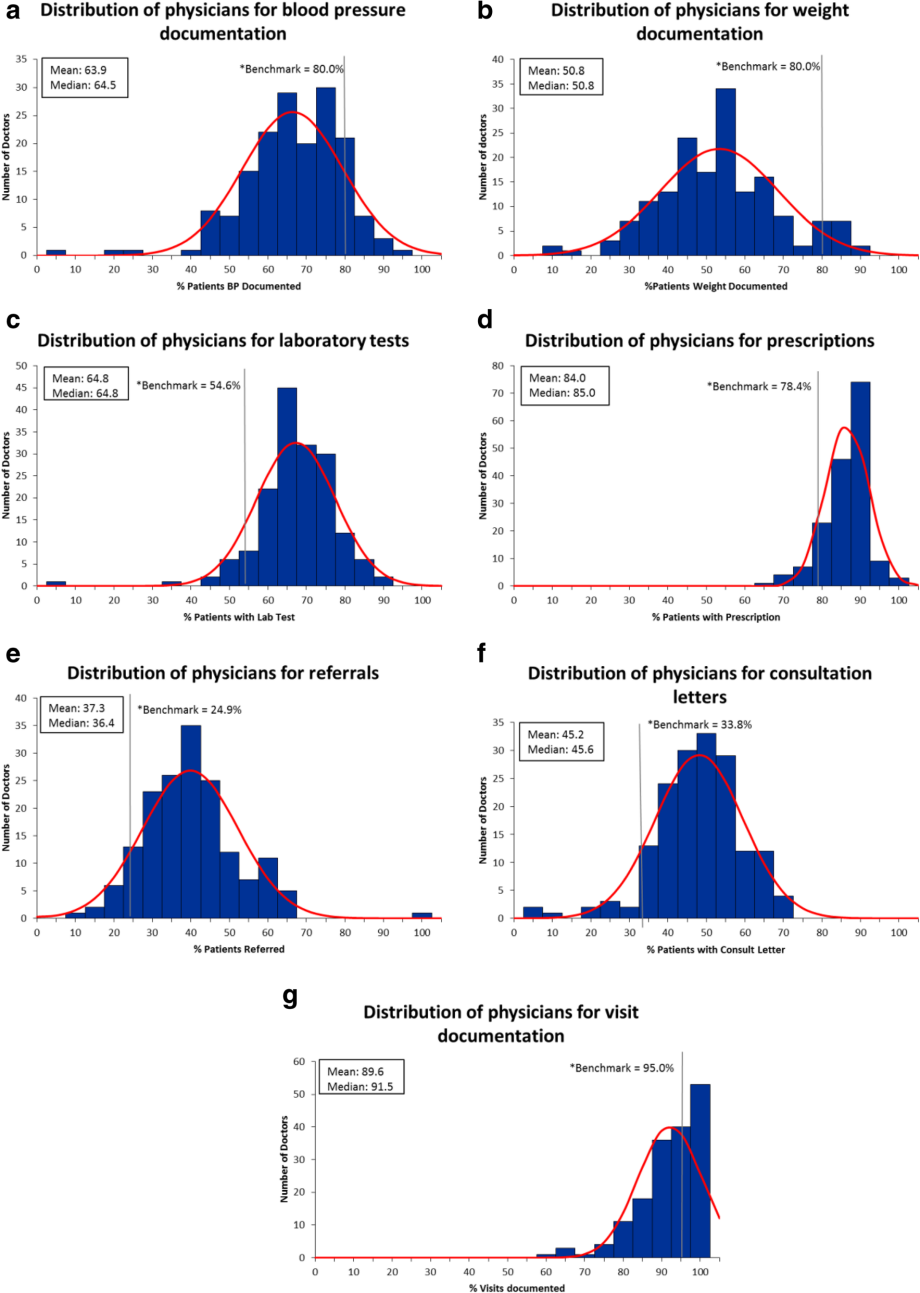
Distribution of physicians for the various EMR fields

**Fig. 5:**
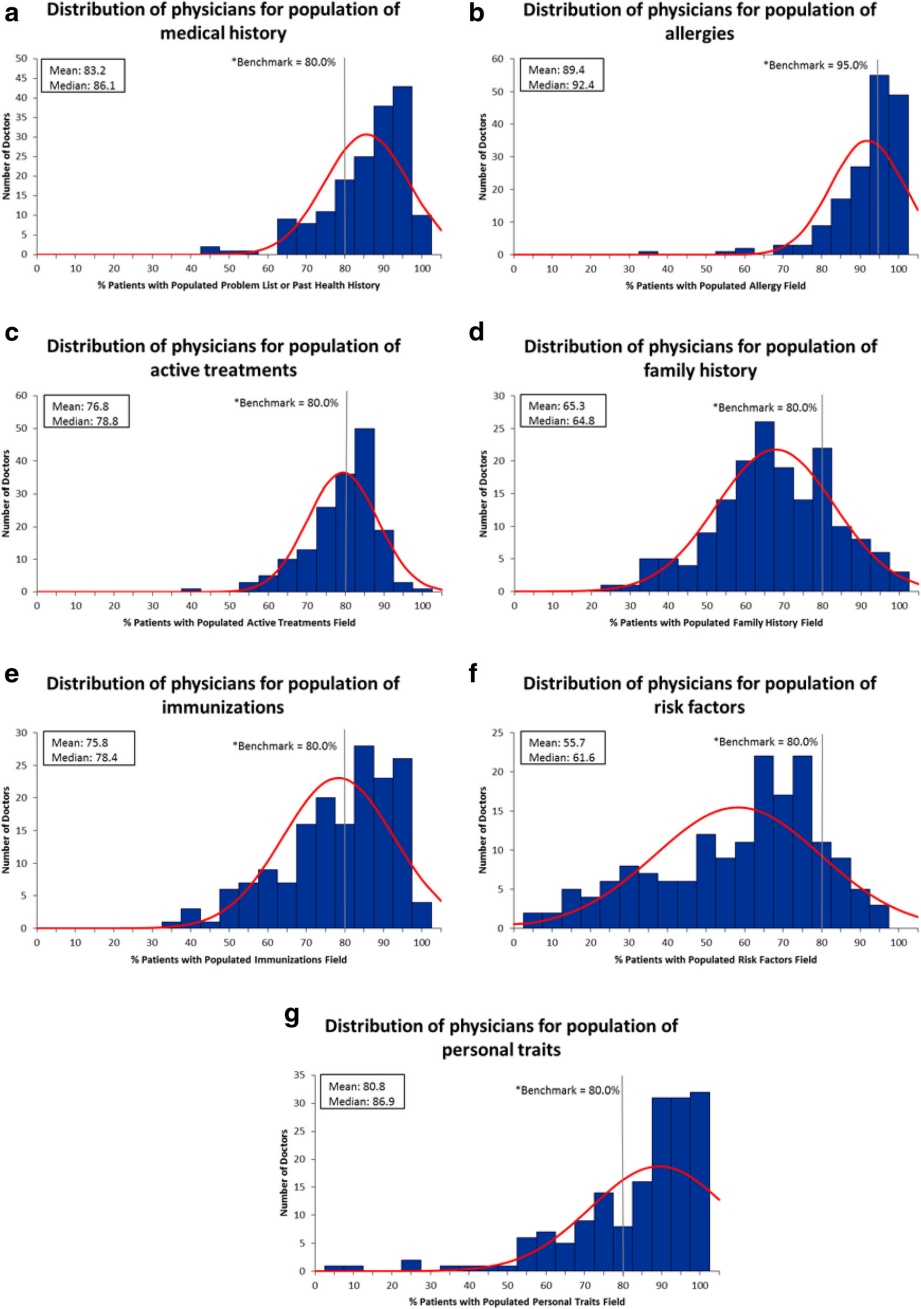
Distribution of physicians for the cumulative patient profile fields

With respect to the frequency distributions for completeness of the fields and the proportion of physicians meeting benchmarks, we found that for fields where benchmarks were set to look for outliers (laboratory tests, prescriptions, referrals and consultation letters) that the majority of physicians exceeded the benchmarks or threshold cut-offs and that outliers or patients with relatively low completion of these fields could easily be identified (Fig. 4). For benchmarks that were set such that the higher the completion the more comprehensive the record we were able to identify areas where completion rates were suboptimal and could be improved upon. The poorest completion of these fields was weight recording, family history and risk factors as evidenced by the majority of physicians falling below the set benchmark (Figs. 4 and 5).

## Discussion

We developed measures to evaluate the extent to which physicians are using the EMR for patient care in Ontario. Overall it appears that physicians are making use of their EMRs within the first year and certainly by the second year post-adoption. Thus, it appears that the current levels of support to use the basic EMR functions are sufficient in achieving adoption of the EMR.

Increased physician and patient duration on the EMR improved data completeness, with the biggest improvements seen between the first and second year. This is similar to a previous study that found recording of preventative screening tests for cancer were not adequately captured in the first year of starting an EMR but improved substantially when looking in the two year window post EMR adoption [18]. Another study found that having two or more years of electronic health record experience was independently associated with reported benefits of having an electronic health record [9]. This time period required for completeness is also relevant to secondary users of the data when making assessment of the quality of care provided or ‘fit for purpose’ assessments, as incomplete EMR data may impede the use of clinical decision support embedded within the EMR, or result in inaccurate analysis of data for quality of care assessment or other types of analysis [28, 29]. Although duration of time on the EMR has not been shown to result in higher performance on quality indicators [30], our study illustrates that both physician and patient time on EMR influence the likelihood of data completeness. The results of this study have led us to develop data quality checks within the EMRALD database to look for outlying physicians to exclude from our studies if a particular field of the EMR is going to be utilized in a study analysis. Our results presented here should have applicability in other settings as EMRALD participants are not just academic sites but draw largely from community practising family physicians.

One of the important features of advanced EMR functions is to automatically generate feedback and reminders at the point of care and to ensure that they are a part of the healthcare providers workflow [31]. Most of these clinical aids rely on completion of EMR data fields in order to accurately report back to the physician. The first step to using advanced functions of the EMR, such as reminders or clinical decision support, is having completed fields for the variables which trigger these tools. While we studied completeness of fields as a measure for EMR usage, we recognize that completeness is only one dimension of data quality [32]. However, assessments of other dimensions of data quality cannot occur without a first assessment of completeness and previous studies looking at the suitability of using EMR data to recruit patients into clinical trials have found data completeness to be an essential component in the assessment of using EMR data for secondary purposes [33]. Whether these completeness measures would hold true with other EMR systems is a potential limitation but we provide real world measures that can be used for comparison with data from other EMR systems.

A previous study has shown that the recording of the presence of some conditions is less frequent in the EMR compared to self-report [34]. Another limitation of this study is that although we were able to determine if there was information recorded in each of the CPP fields we were unable to determine the completeness or quality of recording within each field. Other limitations include the inability to measure the time duration from installment of the EMR to actual usage of the EMR, but given that government funding support is time limited and user fees are charged by EMR vendors from time of installation, it is unlikely that the time from installation to the time of actual use are of significant duration. Another limitation is that we were unable to assess the impact of the local practice environment and/or the presence of allied health professionals on completeness of EMR fields. It is unknown who in a practice was responsible for completing fields such as the CPP or entering in blood pressures. Unfortunately we could also not tell when CPP fields were entered, as we only receive a snap shot of the CPP as it stands on the date of extraction and each item in the CPP is not time stamped. We could also not assess the quality of the data that was entered for example, recording of dates of disease onset. Last, although our patient characteristics were similar to rostered patients in Ontario in terms of presence of chronic diseases and co-morbid conditions, our EMRALD sample of patients were from higher income quintiles and had proportionally more patients living in rural areas compared to the Ontario population. In terms of income quintiles it is not known if this bias is because patients of higher income quintiles may be more likely to seek health care at the primary care level. With respect to a higher proportion of patients residing in rural areas, this proportion constantly changes as new physicians contribute to EMRALD and recruitment is on an ongoing basis. Similarly, our physician characteristics did not exactly match the rest of the family physicians in Ontario. Nonetheless our results showed consistent improvements with completion of fields with increasing duration of time on the EMR despite heterogeneity of physician characteristics.

## Conclusions

In this paper we outline a pragmatic process which we have used to assess completeness of a variety of EMR fields to determine if physicians are adequately using their EMRs and to assess suitability for secondary use. Certainly we know that current EMRs in Ontario are deficient in documenting elements such as hospitalizations and emergency room visits [35]. However, using these described methods in a broad range of EMR fields should be sufficient to allow use of primary care EMR data in a variety of different types of studies as we have employed these methods for data quality checking and have been able to perform a wide range of studies with EMRALD. The types of studies we’ve done have included: assessment of wait times from family physician referral to specialist [36], audit and feedback of quality indicators for chronic disease management [37], validation of administrative data algorithms [38, 39] and within EMR algorithms [40–42] to identify patients with a variety of medical conditions.

Additionally the methods that we have developed here can be used to identify physicians who perform poor on measures of data completeness and are in need of further assistance. Programs could be developed to identify those who fall below their peers and offer support to increase data quality and completeness to get them to more optimal usage. Since we did not examine the use of advanced functions of EMR systems that have been indicated to be important for EMR benefits realization, future work could examine the best point after adoption for them to be implemented, and their impact on patient management, performance on quality indicators and patient outcomes. All users of EMR data, including physicians, administrators, researchers, and policy makers should be acutely aware of the need for understanding data quality and completeness prior to utilizing it for purposes secondary to direct patient care. Furthermore, analysis of both patient time and physician time on EMR data are also important considerations for using EMRs for research as there is a greater likelihood of detecting data with increasing time of a patient’s contribution of data to the database.
